# Arsenic Uptake by Two Tolerant Grass Species: *Holcus lanatus* and *Agrostis capillaris* Growing in Soils Contaminated by Historical Mining

**DOI:** 10.3390/plants9080980

**Published:** 2020-08-01

**Authors:** Agnieszka Dradrach, Anna Karczewska, Katarzyna Szopka

**Affiliations:** 1 Institute of Agroecology and Plant Production, Wrocław University of Environmental and Life Sciences, pl. Grunwaldzki 24a, 50-363 Wrocław, Poland; agnieszka.dradrach@upwr.edu.pl; 2 Institute of Soil Science and Environmental Protection, Wrocław University of Environmental and Life Sciences, ul. Grunwaldzka 53, 50-357 Wrocław, Poland; katarzyna.szopka@upwr.edu.pl

**Keywords:** soil, As, phytoaccumulation, Yorkshire fog, common bent, grassland, fertilization, extractability, translocation factor

## Abstract

The study focused on two grass species *Holcus lanatus* and *Agrostis capillaris* abundant in the sites of former As mining and processing in the Sudetes. Arsenic uptake from soils was examined to assess a risk associated with its accumulation in grass shoots and to check its dependence on soil fertilization. The research involved a field study and greenhouse experiment. In the field study, soil and plant samples were collected from 33 sites with 72–98,400 mg/kg total soil As. Arsenic uptake by grasses differed widely. Both species indicated a strategy typical for eliminators, although As concentrations in more than 50% of the shoot samples exceeded 4 mg/kg, a maximum permissible value for fodder. In the greenhouse experiment, commercial cultivars of both species were grown in five soils containing 394–19,600 mg/kg, untreated and fertilized. All seedlings died in the soil with highest total As, and considerable phytotoxicity was observed in other soils, particularly in nonfertilized ones. Fertilization resulted in the improvement of plant growth and reduction of As uptake except for *Agrostis capillaris* fertilized with manure. Further research should focus on identifying tolerant genotypes growing in extremely enriched sites and analysis of factors that will efficiently reduce As phytoaccumulation.

## 1. Introduction

Arsenic belongs to potentially toxic elements that can be released into the environment from waste materials disposed in the sites of former or contemporary ore mining and processing [1,2,3,4,5,6,7]. In the Polish part of the Sudetes, a mountain range that stretches along the Polish–Czech border, there are three historical arsenic mining centers: Zloty Stok, Radzimowice and Czarnów [8,9], of which Zloty Stok is the largest one. Arsenic was produced there until 1962. Mine wastes and tailings that have remained in those areas contain high concentrations of As, up to 1 percent or even higher [9,10,11,12]. Consequently, the soils that developed in mine sites and in their surroundings are locally highly enriched in As. Some of those soils are now covered with vegetation, some remain barren and others host scattered patches of plants. Improvement of land coverage with vegetation and successful phytostabilization are considered to be the best methods for remediation and management of those areas. Maintaining arsenic concentrations in the aboveground parts of plants at the lowest possible level is therefore one of the crucial aims involved in phytostabilization technique. A permissible concentration of As in animal fodder was set by UE Directive at the level 2–4 mg/kg, depending on the kind of fodder [13]. Environmentalists and researchers use this value as a target concentration of As in the shoots of plants growing in As-contaminated areas, considered safe for potential animal consumers.

Introductory works have indicated that two grass species, *Holcus lanatus* and *Agrostis capillaris*, occur commonly in the areas under study and are sometimes the main components of the local flora. Both species are reported as tolerant to high soil concentrations of arsenic [14,15,16,17,18,19,20], although the mechanisms of tolerance to high soil As are only partly recognized. The data obtained from the literature on As uptake from highly enriched soils and its translocation to the aboveground parts of grass show extremely large variation (Table 1). The concentrations of As in shoots of *Holcus lanatus* and *Agrostis capillaris* can reach 560 mg/kg and 3470 mg/kg, respectively, as shown in the research carried out in the mining areas in England (Cornwall, Devon) and Wales [3,14,21]. However, many other researchers report very low As concentrations in aerial parts of plants, below 5 mg/kg, despite very high As content in soils [22,23,24,25]. The tolerance of examined grass species to very high soil As was not clearly related to exclusion strategy. Tolerant ecotypes were reported as those with both ability to avoid intensive As uptake and as those that accumulate very high As concentrations. This fact prompted plant physiologists and geneticists to take on detailed studies on the mechanisms of tolerance. Such studies, usually carried out in hydroponics, have shown that the tolerance can be achieved mainly by adaptation of the phosphate transport system, leading to reduced influx of arsenate and phosphate into the roots of tolerant plants [14,16,17,18,26,27], which results in reduction of As concentrations in the shoots in comparison with nontolerant genotypes (Table 1). Additionally, it has been proved that normal populations of *Holcus lanatus* can contain the high proportion of As-tolerant genotypes [14,17,26].

It is obvious that the plant uptake of potentially toxic elements depends both on plant species and genotype-related factors, and also on speciation and potential solubility of elements in soils. The release of As from the soil solid phase is determined by its origin and related primary physical and mineralogical forms, by the abundance of As binding components, mainly iron oxihydroxides, and by the presence of components capable to compete with As for specific sorption sites [1,19,20]. Such a competition can be caused by phosphates [33,34,35] or by dissolved or colloidal organic matter [11,36,37,38,39] introduced into the soil solution with inorganic or organic fertilizers.

Inorganic species of As present in soil pore water are generally highly toxic to plants. Arsenate acts as a phosphate analogue and can be easily transported in plants via phosphate cotransport systems [15]. Arsenates can cause generation of reactive oxygen in plants, leading to considerable stress and adverse effects from inhibition of root growth to death. Those effects are particularly strong in nonresistant plant species [15,16,17,18,19,20]. Arsenite is even more toxic to plants as it reacts with sulfhydryl groups (–SH) of enzymes, leading to inhibition of cellular function. It has been proved that conversion of arsenate to arsenite can readily occur in plants. Organic species of As are considered less toxic than inorganic to a wide range of biota, including plants [15]. Soil pH and redox conditions significantly affect As speciation and solubility, and consequently its availability and phytotoxicity in soils. In well-aerated, nonwetland soils, arsenate is normally a highly dominant, or often the only detectable form of As [1].

Numerous works have proved that both phosphates and dissolved organic matter can induce a substantial increase of As concentrations in soil pore water, but this effect will not necessarily result in increased As uptake by plants. On the contrary, it can cause a decrease of As availability due to the competition of phosphates for As uptake by roots. Consequently, the impact of fertilization on As phytoavailability cannot be easily predicted as it depends on the extent of induced As mobilization in soils and phosphate-induced competition for As uptake by roots [33,40]. It is highly dependent on soil and plant properties and each case should be checked experimentally.

The present study involved a greenhouse experiment aimed to assess the effects of inorganic and organic fertilization on the uptake of As by *Holcus lanatus* and *Agrostis capillaris*, and its translocation to the shoots when growing in the soils representative to As mining areas in the Polish part of the Sudetes. Obviously, the conditions of plant growth in the pots and in the field differ considerably [41,42,43], and the results obtained from greenhouse studies cannot be directly compared with those from the field. The major relationships, however, can be more easily recognized and examined under the controlled greenhouse conditions where the impact of uncontrolled factors is usually maintained at a minimum level. Another important aim of the study was to confront the results of the pot experiment with data obtained from the field, in a context of ecological safety for potential animal consumers.

## 2. Materials and Methods

### 2.1. Experimental Areas and Field Study

The study was carried out in sites situated in three historical arsenic mining centers in the Sudetes: Złoty Stok, Radzimowice and Czarnów. More detailed characteristics of their locality were provided in previous papers [9,12,43]. The sites under study included typical hay meadows, wastelands and reclaimed mine waste dumps fully or partly covered by herbaceous and grassy vegetation. Preliminary studies indicated that soils in all those areas contain very high concentrations of As, up to several percent [9]. At the same time, in all the sites examined in this study, the concentrations of other potentially toxic elements remained below those that can pose a serious environmental risk (i.e., below permissible values according to Polish legal regulations, relevant to industrial and mining lands). In each of those areas, several locations were identified with considerable contribution of *Holcus lanatus* or *Agrostis capillaris* in plant cover. Thirty-three such points were chosen for examination. Bunches of one or two grass species in a flowering stage (in June), together with ca. 2 kg lumps of top soil (0–20 cm), were sampled at each point in three replicates. The aboveground parts of grass were cut directly above the crowns while the soil lumps with plant roots were brought to the laboratory where the roots were carefully removed from soil. Soil samples, plant shoots and roots were then prepared for analysis. Large samples of soil material (each of ca. 100 kg), considered representative for different levels of As concentrations and various sources of soil enrichment in As, were collected from the topsoil (0–20 cm) at five sites. Soil material was sieved on-site to pass a 20 mm screen, transported to the greenhouse and prepared for the pot experiment.

### 2.2. Pot Experiment

Soil material was air-dried, homogenized, sieved through a 5 mm screen, and divided into three parts of which one was left untreated (0) while two others were fertilized with a solution containing the mixture of inorganic fertilizers (F) at the rate of 250 mg/kg NPK (including 50 mg/kg P), or with granulated cattle manure (M) at the rate 10 g/kg. The composition of fertilizing solution and the properties of manure were described in more detail in the previous paper [43]. An unpolluted sandy loam soil that contained 3.6 mg/kg As was used as control (C) and was treated in like manner. Soil material was homogenized, moistened with distilled water and stabilized for 2 weeks at a moisture level of ca. 70% of water-holding capacity (WHC) prior to the pot experiment. After that time, soil material was put into 1 kg plastic pots supplied with drainage, placed in the greenhouse and sown with commercial cultivars of *Holcus lanatus* and *Agrostis capillaris* (ca. 50 and 120 pcs. per pot, respectively). The experiment was carried out in three replicates. The pots were watered regularly to maintain soil moisture close to 70% of WHC. After 10 weeks of growth, grass shoots were harvested above the crowns. Plant roots were then separated from the soil. All the samples of plant material were thoroughly washed with tap water and deionized water, oven-dried for 48 h at 60 °C, weighted and ground. Soil material was air-dried and prepared for analysis.

Air-dried soil samples from the field and from the greenhouse study were homogenized, ground, sieved to 2 mm and analyzed using standard methods applied in soil science, as described previously [12,43]. “Pseudototal” concentrations of As in soils (called “total”), were determined after microwave soil digestion with aqua regia (HNO_3_ + HCl), following ISO 14466. Easily soluble As species were extracted from soils with 1 M NH_4_NO_3_ (*m*:*v* 1:2.5, 2 h). The potentially soluble pool of soil phosphorus, considered phytoavailable, was determined by a lactate/Ca method [44], a standard procedure used for agricultural purposes in Poland. The concentrations of As and P in digests and extracts were measured by ICP–AES, with determination limits at 0.005 mg/L and 0.02 mg/L, respectively. Determination of total As was validated with two certified soil materials, CNS 392 and CRM 027, and the accuracy of As determination in NH_4_NO_3_ extracts was checked by standard addition.

The concentrations of As and P in plant material were determined by ICP–AES, as described above, after sample pretreatment with 30% H_2_O_2_ and microwave digestion in concentrated HNO_3_. Analytical methods were validated with plant reference materials: BCR-414 and DC-7349.

### 2.3. Data Interpretation and Statistics

Interpretation of data involved the calculations of transfer, bioaccumulation and bioconcentration factors (TF, BAF and BCF) that were defined and discussed in details in the previous papers [12,43]. Briefly, TF was calculated as the ratio of As concentration in grass shoots to roots. Two other factors were determined as the ratios between As concentration in plant biomass (shoots or roots) and total (BAF) or extractable (BCF) As in soil. The index of toxicity (ToxI) that illustrates plant response to adverse soil factors was calculated based on the pot experiments, by comparing the shoots biomass of grass grown in As-rich soil and in control soil (C) fertilized analogously.

The least statistically significant differences between various treatments of the pot experiment were calculated by Fisher’s test, at *p* < 0.05, using Statistica 13.0 (StatSoft Polska, Kraków, Poland) software. The graphs that illustrate the relationships between plant and soil data, and between various treatments in the pot experiment were produced with Excel 2010 (Microsoft).

## 3. Results and Discussion

### 3.1. Soil Properties

Summarized data that illustrate the properties of soil samples collected in the field and those used in the greenhouse experiment are presented in Table 2. Detailed data from the field are listed in the Supplementary Materials (Table S1). Total As in all soils examined ranged from 72 to 98,400 mg/kg, while soil material collected for the greenhouse study contained As in concentrations that ranged from 394 to 19,600 mg/kg. The amounts of 1M NH_4_NO_3_-extractable As in those soils were relatively low, in the range 0.18–12.9 mg/kg. Soil 5, representative of a reclaimed mine dump called “Orchid Dump” [12,43], had the highest concentration of total As, while the highest concentration of extractable As was present in the soil 2, representative of dry meadow on the plateau built of tailings.

### 3.2. Plant Growth in Greenhouse

**Figure 1:**
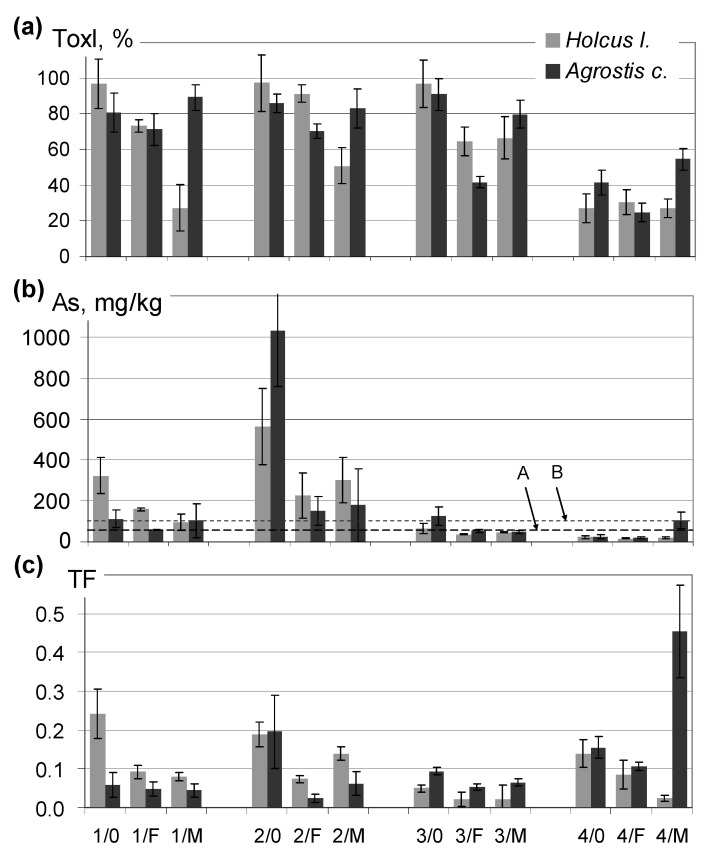
The results of a pot experiment. Parameters that illustrate the growth of grass and As uptake from soils: (**a**) toxicity index ToxI, %, calculated based on the difference between plant biomass in the soil examined and analogously treated control soil; (**b**) As concentration in plant shoots, A and B, stand for maximum As concentrations in the shoots of *Holcus lanatus* and *Agrostis capillaris,* respectively, growing in the field; (**c**) As translocation factor. Error bars stand for the least significant difference based on three replicates. Explanations: ToxI—toxicity index; TF—root to shoot translocation factor; 1, 2, 3, 4—soils used in the pot experiment; 0, F, M—various fertilization methods: 0—no fertilization, F—inorganic fertilization, M—fertilization with manure.

The growth of plants in the greenhouse experiment differed greatly among the soils and was strongly dependent on fertilization. Germination of both grass species sown in the soil 5 was very poor, and the seedlings died shortly after appearance, so that the analysis of plant material could not be performed. In all the remaining soils, seed germination and the growth of grass were assessed as satisfactory, but the biomass obtained from the experiment differed strongly among the treatments. Figure 1a presents the values of toxicity index ToxI calculated for soils 1–4 based on the difference of plant biomass obtained from the soil examined and from analogously treated control soil (unpolluted). The ToxI index was the highest, above 95%, in nonfertilized soils 1–3, but fertilization resulted in its significant reduction in most cases, which means that differences between the biomass obtained from polluted and unpolluted soils narrowed considerably due to application of fertilizers. In the case of soil 4, with the lowest total As concentration (394 mg/kg), the toxicity effects observed for both grasses, particularly for *Holcus lanatus*, were much weaker, and the values of ToxI determined for the nonfertilized soil 4 remained below 50%. The value of ToxI, determined for soil 5 was, of course, 100%.

The two grass species examined in the pot experiment turned out to react differently to fertilization. Application of manure had a beneficial effect on the growth of the Yorkshire fog (*Holcus lanatus*) in strongly contaminated soils 1, 2 and 3, while no improvement of the growth was observed for common bentgrass, *Agrostis capillaris*. In the case of soil 4, manure adversely affected the growth of the latter grass. Soil treatment with inorganic fertilizers (F) proved to be particularly beneficial for *Agrostis capillaris* grown in soils 1 and 3 (Figure 1a). Considerable improvement of the growth of grasses resulting from soil fertilization, and associated reduction in As concentrations in grass shoots (Figure 1b, discussed further in the text), should be emphasized, because fertilization of As-rich soils, especially application of organic fertilizers, results usually in an accelerated release of As from the soil solid phase, increase in its solubility and elevated As concentrations in soil pore water, which were documented in earlier studies [11,34,38,43].

### 3.3. Arsenic Uptake from Soils in the Greenhouse

Increased As solubility (Figure S1) and its undoubtedly increased concentrations in soil pore water did not cause an enhanced uptake of As by grass and translocation to the shoots in most of the experimental treatments. The plants grown in fertilized soils usually had significantly lower concentrations of As in their aerial biomass compared to nonfertilized ones, except for *Agrostis capillaris* grown in M-amended soil 4, the case mentioned above. Similar effects were reported from several other studies [18,33,35,45] and explained by a high P concentration in the pore water of fertilized soils and a competition between P and As for the uptake by roots. The opposite effect, i.e., an increased As uptake by plants from fertilized As-contaminated soils, was also reported by several authors [46,47].

**Figure 2:**
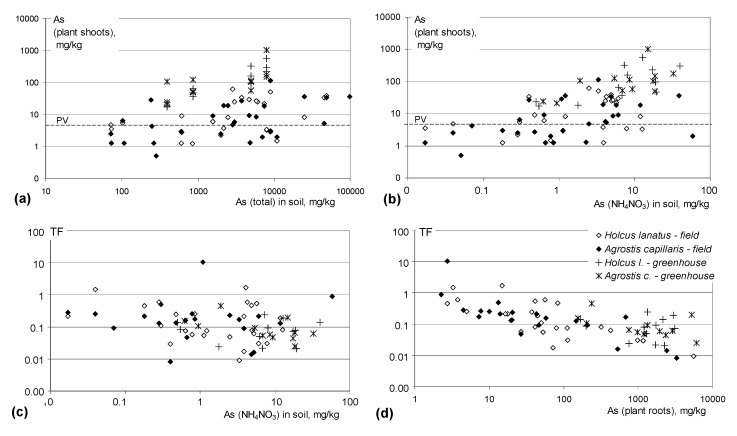
As concentrations in the aboveground parts of *Holcus lanatus* and *Agrostis capillaris*, and the values of translocation factors TF in the field survey and in greenhouse experiment. (**a**) Concentrations of As in grass shoots related to total As concentrations in soils; (**b**) Concentrations of As in grass shoots related to 1 M NH_4_NO_3_-extractable As in soils; (**c**) As translocation factors TF in relation to 1M NH_4_NO_3_-extractable As in soils; (**d**) As translocation factors TF in relation to As concentrations in grass roots. PV—permissible value for dry fodder (4 mg/kg).

The concentrations of As in plants were clearly related to the biomass of grass (Figure 1b). The highest As concentrations in both *Holcus* and *Agrostis* aboveground biomass, exceeding 500 and 1000 mg/kg, respectively, were found in the nonfertilized soil 2, which developed on pure tailings that had the largest share of extractable As. Such high As concentrations in grass biomass were much higher that those found in the field study, as shown in Figure 1b and Figure 2.

### 3.4. Arsenic Uptake in the Field

The median values of bioaccumulation factor BAF calculated for the shoots of grass samples collected from the field were very low (BAF < 0.01) (Table 3), proving that both *Holcus lanatus* and *Agrostis capillaris* follow the strategy typical for As excluders. The median concentrations of As in their aboveground parts (7.1 and 5.2 mg/kg, respectively), however, were higher than 4 mg/kg, which means that more than 50% of collected grass samples should be considered potentially hazardous for animals. Detailed data on As concentrations in plant material collected from the field, as well as related TF, BAF and BCF factors, are provided in Supplementary Materials (Table S2).

The concentrations of As in plant shoots collected from the field did not depend on total concentrations of As in soils, which can be easily seen from Figure 2a. Related coefficients of determination *R*^2^ were very low (<0.04). Even at very high soil As concentrations, at the level of 10,000 mg/kg, As concentrations in the shoots of both grass species can remain below the permissible value PV = 4 mg/kg. On the other hand, however, the cases of As concentrations in plant shoots above 4 mg/kg were reported from the sites with relatively low soil total As of ca. 100 mg/kg.

The analysis of relationships between As concentrations in grass shoots and 1M NH_4_NO_3_-extractable As in soils (Figure 2b) shows that visible, though insignificant at *p* < 0.05, correlations between them exist, and can be particularly well seen in the range of extractable As above 0.1 mg/kg. One could draw a conclusion that increased solubility of As in soils, which can be caused by fertilization or decomposition of organic matter, would bring about an increase in As concentrations in grass shoots. However, our results obtained from the pot experiment showed an opposite effect. Soil fertilization improved considerably the growth of grass causing at the same time a decrease in As concentrations in plants rather than their increase.

### 3.5. Comparison of Greenhouse and Field Data

The analysis of data obtained from the field study and the greenhouse proved clearly that As concentrations in plants in the pot experiment differed from those in the field. Compared cases with the same total As (Figure 2a), or the same extractable As (Figure 2b), indicate that the concentrations of As in biomass of grass grown in the pot experiments were much higher than those in grass sampled from the field. This effect can be partly caused by the specificity of pot experiments in which the conditions of plant growth are different from the field [41,42]. However, the considerably low As uptake by grass growing in the field may very likely be associated with tolerant genotypes capable to reduce the influx of As to the roots and minimize its further transport to the aerial parts of plants. This phenomenon was described by Porter and Peterson [14] and further examined by Macnair, Meharg and their coworkers [15,16,17,18,48]. It is worth mentioning that the concentrations of P in grass shoots in our study were also significantly higher in the pot experiments than those in field samples (Table 3). Significant differences were confirmed between plant material obtained from the field and from nonfertilized treatments in the pots (detailed data are not presented). Soil fertilization in the pots resulted obviously in an increase in P supply in soils and increased P uptake by plants; therefore, the median values of P concentrations in plant biomass, presented in Table 3, were much higher in the pot experiment than those obtained from the field where fertilization was carried out occasionally, if at all. An increase in P uptake by plants caused by fertilization was most pronounced in greenhouse soils 1 and 3, which differed considerably in their pools of available P (Table 2). This effect and the factors that caused different uptake of P by plants seem to be worth closer analysis and discussion in another paper. At this stage of our research, no clear relationships were found between the ratio of soluble P/As and the parameters of As uptake (Figure S2).

The values of As translocation factor TF did not actually depend on soluble As in soil and remained in similar ranges in the field samples and in the pot experiment (Figure 2c). For both grass species, TF values determined in the field tended to decrease with increasing As concentration in plant roots, particularly in the range of very high As root concentrations, above 1000 mg/kg (Figure 2d). This tendency may be explained as the effect of particular tolerance in the conditions of extreme As concentrations in soils, when the transport of As to the aboveground parts of grasses does not run passively, and that the mechanisms that block As translocation from the roots to aerial parts of grasses are involved [20,26,27]. At similarly high concentrations of As in roots of grass grown in the pot experiment, clearly higher TF values were observed, which may indicate the lack of such barriers in commercial nontolerant or poorly tolerant grass genotypes.

The data presented in Table 3 provide a summary of results obtained from the field study and greenhouse experiment. The median values of As concentrations in plant biomass were for both species much higher in the pot experiment than those in the field despite the fact that total As in soil 3 and 4 was much below the median value of total As in field soils. Generally, As concentrations in the shoots of grass growing in the field were far lower than those reported for *Agrostis capillaris* by Porter and Peterson [3,14] and Benson et al. [21] from English and Welsh mining areas, where they exceeded 1000 mg/kg. Perhaps such high values should be now verified with validated analytical methods, more advanced than those used over 40 years ago (Table 1). On the other hand, however, our pot experiment proved that plants of both species examined can accumulate very high concentrations of As in their shoots, of several hundred mg/kg, without visible signs of phytotoxicity. Therefore, the research focusing on the uptake of As by plants growing in the areas of highly enriched As should be continued, both in the areas that were subjected to natural succession and in those that will be managed to support phytostabilization. Particular issues that should be considered therein are (1) the factors that determine As accumulation in plant shoots and a related hazard to potential animal consumers, and (2) identification of tolerant genotypes and their response to fertilization. Differences between commercial cultivars and potentially tolerant ones should be examined in the future by using a seed material from the field ecotypes in pot experiments and by using the commercial seed material in the field.

## 4. Conclusions

Both species of grass were able to grow in soils highly enriched in arsenic in former mine sites; however, the greenhouse experiment proved considerable phytotoxicity of those soils, particularly when they remained nonfertilized. The properties of mine dump material with extremely high As concentrations can even be lethal for plant seedlings derived from commercial seed.

Basically, both *Holcus lanatus* and *Agrostis capillaris* indicated an As uptake strategy typical for excluders, with TF below 1, though the concentrations of As in the shoots of more than 50% of the samples growing in the field exceeded 4 mg/kg, a maximum permissible value for animal fodder.

The uptake of As from soils proved to depend on various factors, including As extractability from soils and the fertilization applied. Soil treatment with both inorganic and organic fertilizers resulted in an improvement of plant growth and usually caused a substantial reduction of As concentrations in the aboveground parts of grass. A likely increase in As concentrations in grass shoots due to soil manuring should also be considered, however, particularly in the case of *Agrostis capillaris*.

Further research should focus on identifying tolerant genotypes of grasses that grow in the sites with extremely high As concentrations in soils, as well as on thorough examination of factors that can efficiently reduce As uptake and its accumulation in the aboveground parts of plants.

## Figures and Tables

**Table 1:** Concentrations of As in the roots and shoots of *Holcus lanatus* and *Agrostis capillaris* reported from soils enriched in As or from As-supplied hydroponics.

As in Soil mg/kg	As in Roots mg/kg	As in Shoots mg/kg	Origin of Data	Details on the Site or Experiment	Analytical Method	Source
***Holcus lanatus***						
n.d. ^1^	n.d.	Mean 350 Max. 560	Field (mine sites)	Rural area, high As sites, Tolvaddon Downs	Acid digestion (HNO_3_ + H_2_SO_4_), arsine generation, colorimetric determination Ag–DDC^2^	[3]
128–130 (0–10 cm)	n.d.	0.73	Field (mine sites)	El Cabaco mine soils	Digestion in aqua regia + HF, AAS	[22]
595–2568 (0–25 cm)	n.d.	0.61	Field	A medieval silver mine—Kutna Hora (Czech Republic)	Dry ashing, HG–AAS	[23]
Up to 17,000; water soluble As: 0.2–2.1 mg/kg	n.d.	28–158	Field plot experiment: (54 × 20 m)	Various kinds of treatment for remediation	Digestion in mixed acid (HNO_3_:HCl, 4:1), HG–AAS	[28]
1325 (0–30 cm)	2030–2980	130–166	Pot experiment	Soil of old mine Jales amended with industrial sugar residue	Digestion in mixed acid (HNO_3_:HCl, 4:1), HG–AAS	[29]
	2.2–22	2.0–6.7	Hydroponic study, 24 h exposure	1 mg/L As in various forms	Digestion in concentr. HNO_3_ + H_2_O_2_	[27]
	10–300	5–30	Hydroponic study, 30 d exposure	0.1 mg/L As	HNO_3_ digestion, Hydride generation, ICP–MS	[26]
	40–1400	20–45		0.6 mg/L As		
	300–7400	140–950		8 mg/L As		
***Agrostis capillaris***						
n.d	n.d.	Mean: 1480, Max 3470	Field, mine sites (high arsenic sites)	Mine wastes sites (Gawton Utd. Mine)	Digestion in HNO_3_ + H_2_SO_4_, arsine generation, colorimetric determination Ag–DDC	[3]
n.d.	n.d.	Mean: 1.4, Max. 3	Field (low arsenic sites)	Westfield College, London, reference		
8510–26,530 Median: 12,800	n.d.	3–3470; Median: 430	Field, mine-affected sites	Various mine-affected sites and reference soil	Digestion in HNO_3_ + H_2_SO_4_, arsine generation, colorimetric determination Ag–DDC	[14]
	n.d.	Mean: 675 (90–2080)	Field	Wheal Josiah		
	n.d.	Mean: 1070 (90–3470)		Gawton Utd. Mine		
20 (?)	n.d.	1.4–3.0		Westfield College, London, reference		
630	nd	1.1	Field, tailings	A surface of tailings pond	Digestion in mixed acid (HNO_3_, H_2_SO_4_ + HCIO_4_), HG–AAS	[24]
8–300	n.d.	0.2–8	Field ^3^	Various industrial sites in Flanders	Acid digestion, high-resolution ICP–MS	[30]
395–480 (0–2.5 cm) 76 (2.5–5.1 cm)	34–58	33–35	Pot and field plot experiments	Application of calcium arsenate as herbicide	Official method of the Assoc. of Agricultural Chemists. USA, 1955	[31]
3160 +/− 800	1040	280	1-month culture greenhouse experiment	Polluted soil, industrial wasteland of Auzon	Digestion in concentr. HNO_3_ + H_2_O_2_, GF–AAS	[32]
234 +/− 46	34	5		Naturally enriched soil (hydrothermal deposits)		
203	n.d.	1.4	Semi-field experiment (large pots)	Mine-contaminated soil, Aljustrel mining area	Dry ashing, FAA/GF–AAS	[25]
	15 (T ^4^), 26 (N ^4^)	n.d.	Hydroponic, 40 min. exposure	3.7 mg/L; tolerant plants from Devon Great Console mine	Digestion in concentrated HNO_3_, HG–AAS	[16]
	105 (T) 570 (N)	35 (T) 75 (N)	Hydroponic, 7 d exposure	5 mg/L	Digestion in HNO_3_+H_2_SO_4_, arsine generation, colorimetric determination Ag–DDC	[14]
	1305–2880 (T) 1960–3320 (N)	230–565 (T) 240–875 (N)		20–50 mg/L		
	6950–7700 (T) 4930–8890 (N)	445–795 (T) 555–1625 (N)		100–150 mg/L		

^1^ n.d.: no data; ^2^ Ag–DDC: silver diethyl dithiocarbamate; ^3^ Mixed species: *Agrostis c.* + *Poa* sp.; ^4^ T: tolerant, N: nontolerant genotypes.

**Table 2:** Properties of soils used in the greenhouse experiment (mean values of five replicates) and examined in the field study.

Soil/Site	Description	Clay, %	Textural Group ^1^	Corg, %	pH	As Total mg/kg	As (NH_4_NO_3_) mg/kg	P Available mg/kg
1	Złoty Stok, hay meadow flooded with tailings	6	SL	2.45	6.38	5020	7.32	242
2	Złoty Stok, plateau built of tailings, dry meadow	3	LS	0.25	7.40	8000	12.9	194
3	Złoty Stok, hay meadow flooded with tailings	1	LS	1.89	5.68	856	3.86	38
4	Radzimowice, hay meadow, the vicinity of former mines and smelter	8	L	1.97	4.18	394	0.18	18
5	Złoty Stok, reclaimed mine dump, “Orchid Dump”	3	LS	0.13	5.50	19,600	3.61	109
All fields (*n* = 33)	Min	0	x	0.20	2.88	72	0.02	6
	Median	3	x	1.70	4.84	3030	1.22	41
	Max	9	x	17.2	7.60	98,400	58.5	290

^1^ Textural groups according to U.S. Department of Agriculture.

**Table 3:** Summary of As concentrations in plant biomass and plant-related indices (transfer, TF; bioaccumulation, BAF; bioconcentration, BCF) determined in the greenhouse experiment and in the field.

Soil/Site		As in Shoots mg/kg		As in Roots mg/kg		TF	BAF Shoots	BAF Roots	BCF Shoots	BCF Roots	P (g/kg) Shoots	P (g/kg) Roots
		Median	(Max)	Median	(Max)	Median						
***Holcus lanatus***												
1		158	(323)	1340	(1730)	0.09	0.03	0.27	20	183	1.63	1.34
2		300	(561)	2990	(3110)	0.14	0.04	0.37	13	179	3.25	2.98
3		47	(65)	1710	(2260)	0.02	0.05	2.00	5	210	3.14	1.17
4		18	(23)	201	(760)	0.08	0.05	0.51	32	372	3.65	2.25
All fields (*n* = 26)	Min	1.2		2.8		0.01	<0.001	0.001	0.3	3	0.41	0.52
	Median	7.1		52		0.10	0.004	0.012	7	41	0.69	0.72
	Max	62		5570		1.70	0.06	0.63	206	2900	1.25	1.39
***Agrostis capillaris***												
1		103	(113)	1940	(2340)	0.05	0.02	0.39	6	132	3.91	1.33
2		180	(1030)	5250	(6100)	0.06	0.02	0.66	8	330	4.52	3.38
3		53	(124)	980	(1330)	0.06	0.06	1.15	8	142	3.56	1.51
4		24	(107)	202	(237)	0.15	0.06	0.51	39	215	5.35	2.11
All fields (*n* = 29)	Min	0.5		2.3		<0.01	<0.001	<0.001	<0.1	1	0.48	0.59
	Median	5.2		44		0.16	0.002	0.05	5	69	1.02	1.19
	Max	115		9400		10.3	0.12	0.89	74	8290	1.48	1.73

## Supplementary Materials

The following are available online at https://www.mdpi.com/2223-7747/9/8/980/s1. Figure S1: Concentrations of 1 M NH_4_NO_3_-extractable As in soils in the pot experiment, Figure S2. Relationships between As concentrations in plant shoots (a) and the values of TF (b) vs. a ratio of soluble P/As in soils, Table S1. Detailed data on the properties of soils examined in the field study in sites P01-P33, Table S2. Detailed data on As concentrations in plant biomass and plant-related indices, determined in the field.
