# Supplementary material for: Arsenic Uptake by Two Tolerant Grass Species: Holcus lanatus and Agrostis capillaris Growing in Soils Contaminated by Historical Mining
Source: Plants (Basel). 2020 Aug 1;9(8):980. doi: 10.3390/plants9080980 (PMC7464124; doi:10.3390/plants9080980)
Supplement: Supplementary file 1 [file plants-09-00980-s001.pdf]

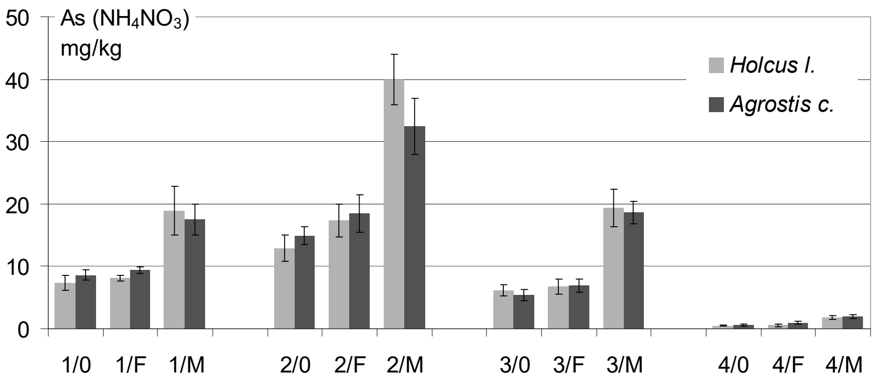

Figure S1. Concentrations of 1M NH_4NO_3-extractable As in soils in the pot experiment.

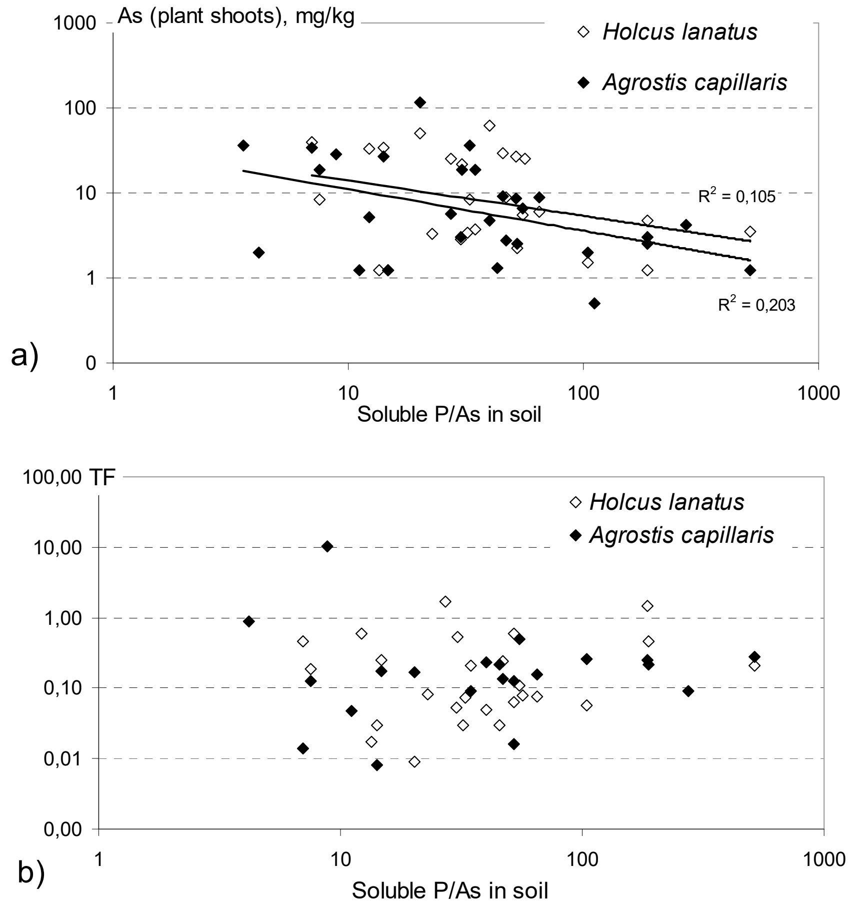

Figure S2. Relationships between As concentrations in plant shoots (a) and the values of TF (b) vs. a ratio of soluble P/As in soils.

Table S1. Detailed data on the properties of soils examined in the field study in sites P01-P33. The data are mean values of 3 replicates.

Site No	Clay content %	Corg %	pH	As total mg/kg	Soluble As. mg/kg	Soluble P. mg/kg	Soluble P / As
P01	1	1.39	5.81	8900	3.32	67	20
P02	3	0.59	4.66	48910	4.88	34	7
P03	4	1.58	4.82	1566	0.63	41	65
P04	5	3.95	6.66	6069	4.96	281	57
P05	3	4.75	6.6	5836	5.21	270	52
P06	2	3.22	6.55	4738	6.04	275	46
P07	3	4.18	6.19	2840	2.49	100	40
P08	1	2.12	4.81	607.8	0.48	22	46
P09	2	1.88	5.6	2174	3.86	134	35
P10	0	0.85	4.84	106.7	0.85	13	15
P11	2	1.7	3.53	102	0.3	17	57
P12	4	3.34	3.59	11000	0.78	81	104
P13	5	11.83	4.01	2477	11.88	90	8
P14	7	2.31	3.93	265	0.67	7	10
P15	9	2.16	4.39	248	0.07	19	271
P16	8	1.96	3.6	603	0.18	34	189
P17	9	17.2	3.91	73	0.02	9	450
P18	6	1.28	3.99	72	0.04	7	175
P19	5	0.82	7.4	7040	58.52	244	4
P20	5	4.11	4.44	2005	0.28	15	54
P21	3	3.5	3.72	243.2	1.09	10	9
P22	4	4.17	3.98	283.8	0.05	6	120
P23	5	1.6	2.88	98400	38.68	138	4
P24	6	1.38	5.03	45600	4.25	52	12
P25	2	0.201	3.72	3750	0.4	6	15
P26	5	2.35	4.72	25100	1.22	40	33
P27	4	0.82	4.46	8900	1.13	34	30
P28	3	0.49	7.6	8000	12.7	290	23
P29	3	0.72	7.22	7950	7.85	253	32
P30	3	1.62	5.88	4830	1.21	99	82
P31	2	1.54	5.41	3026	4.12	112	27
P32	3	1.69	5.68	850	3.86	41	11
P33	3	0.89	7.18	7417	5.69	174	31

Table S2. Detailed data on As concentrations in plant biomass and plant-related indices, determined in the field.

Site No	Total As concentrations, mg/kg		TF	BAF		BCF (related to NH_4NO_3-extractable As)	
	shoots	roots		shoots	roots	shoots	roots
Holcus lanatus							
P1	50,8	5568	0,01	0,010	0,63	15,3	1677
P2	39,0	83,8	0,47	0,000	0,00	8,0	17,2
P3	6,0	79,5	0,08	0,000	0,05	9,5	125
P4	25,0	318	0,08	0,000	0,05	5,0	64,0
P5	26,5	425	0,06	0,000	0,07	5,1	81,5
P6	29,3	978	0,03	0,010	0,21	4,8	162
P7	61,5	1249	0,05	0,020	0,44	24,7	502
P8	9,0	37,3	0,24	0,010	0,06	18,9	78,3
P9	3,8	17,8	0,21	0,000	0,01	1,0	4,6
P10	1,3	5	0,25	0,010	0,05	1,5	5,9
P11	5,5	50,3	0,11	0,050	0,49	18,3	167
P12	1,5	26,5	0,06	0,000	0,00	1,9	34,0
P13	8,3	44,5	0,19	0,003	0,02	0,7	3,7
P16	1,3	2,8	0,45	0,004	0,00	7,0	15,3
P17	3,5	16,5	0,21	0,050	0,23	206,0	971
P18	4,8	3,3	1,46	0,067	0,05	119,0	81,8
P20	2,3	3,8	0,60	0,005	0,00	7,9	13,2
P24	33,6	56,2	0,60	0,000	0,00	7,9	13,2
P25	34,3	1163	0,03	0,010	0,31	85,6	2908
P26	8,3	112	0,07	0,005	0,00	6,8	91,5
P27	2,9	53	0,05	0,000	0,01	2,5	46,9
P28	3,3	41,1	0,08	0,000	0,01	0,3	3,0
P29	3,4	114	0,03	0,000	0,01	0,4	14,5
P31	25,4	14,9	1,70	0,010	0,00	6,2	3,6
P32	1,2	71,6	0,02	0,000	0,08	0,3	18,5
P33	22,1	41,4	0,53	0,000	0,01	3,9	7,3
Agrostis capillaris							
P1	115,0	683	0,17	0,010	0,08	34,6	206
P2	33,8	2417	0,01	0,000	0,05	6,9	496
P3	9,0	57,5	0,16	0,010	0,04	14,2	90,7
P5	8,5	536	0,02	0,003	0,09	1,6	103
P6	9,3	43,5	0,21	0,002	0,01	1,5	7,2
P7	4,8	20,5	0,23	0,000	0,01	1,9	8,2
P8	2,8	20,3	0,14	0,000	0,03	5,8	42,6
P9	19,0	210	0,09	0,010	0,10	4,9	54,5
P10	1,3	7,3	0,17	0,010	0,07	1,5	8,5
P11	6,5	13,3	0,49	0,060	0,13	21,6	44,0
P12	2,0	7,8	0,26	0,000	0,00	2,6	10,0
P13	18,8	147	0,13	0,010	0,06	1,6	12,4
P14	1,3	26,5	0,05	0,000	0,10	1,9	39,8
P15	4,3	46,8	0,09	0,020	0,19	60,5	665
P16	3,0	14	0,21	0,000	0,02	16,7	78,0
P17	1,3	4,5	0,28	0,020	0,06	73,5	265
P18	2,5	10	0,25	0,030	0,14	62,9	252
P19	2,0	2,3	0,89	0,002	0,00	0,0	1,0
P20	2,5	19,5	0,13	0,000	0,01	8,8	69,0
P21	28,3	2,8	10,3	0,120	0,01	26,0	2,5
P22	0,5	2,3	0,22	0,000	0,01	9,9	44,3

P23	36,0	9400	0,00	0,002	0,10	0,9	243
P24	5,2	2101	0,00	0,003	0,05	1,2	494
P25	27,0	3318	0,01	0,010	0,88	67,5	8290
P26	36,3	4264	0,01	0,000	0,17	29,7	3490
P27	3,0	170	0,02	0,000	0,02	2,7	150
P30	1,3	207	0,01	0,000	0,04	0,6	90,5
P31	5,7	14,9	0,38	0,000	0,00	1,4	3,6
P33	18,5	75,5	0,25	0,000	0,01	3,2	13,3
